# HisAK70: progress towards a vaccine against different forms of leishmaniosis

**DOI:** 10.1186/s13071-015-1246-y

**Published:** 2015-12-09

**Authors:** Gustavo Domínguez-Bernal, Pilar Horcajo, José A. Orden, José A. Ruiz-Santa-Quiteria, Ricardo De La Fuente, Lara Ordóñez-Gutiérrez, Abel Martínez-Rodrigo, Alicia Mas, Javier Carrión

**Affiliations:** INMIVET, Department of Animal Health, Faculty of Veterinary Science, Complutense University of Madrid, Madrid, 28040 Spain; SALUVET, Department of Animal Health, Faculty of Veterinary Science, Complutense University of Madrid, Madrid, 28040 Spain; “Severo Ochoa” Molecular Biology Centre CSIC-UAM, Madrid, 28049 Spain

**Keywords:** *Leishmania infantum*, *Leishmania major*, Cross-protection, Leishmaniosis, Vaccine, DNA, Histones, A2, Kmp11, Hsp70, Granuloma

## Abstract

**Background:**

*Leishmania major* and *Leishmania infantum* are among the main species that are responsible for cutaneous leishmaniosis (CL) and visceral leishmaniosis (VL), respectively. The leishmanioses represent the second-largest parasitic killer in the world after malaria. Recently, we succeeded in generating a plasmid DNA (pCMV-HISA70m2A) and demonstrated that immunized mice were protected against *L. major* challenge. The efficacy of the DNA-vaccine was further enhanced by the inclusion of KMP-11 antigen into the antibiotic-free plasmid pVAX1-asd.

**Methods:**

Here, we describe the use of a HisAK70 DNA-vaccine encoding seven *Leishmania* genes (H2A, H2B, H3, H4, A2, KMP11 and HSP70) for vaccination of mice to assess the induction of a resistant phenotype against VL and CL.

**Results:**

HisAK70 was successful in vaccinated mice, resulting in a high amount of efficient sterile hepatic granulomas associated with a hepatic parasite burden fully resolved in the VL model; and resulting in 100 % inhibition of parasite visceralization in the CL model.

**Conclusions:**

The results suggest that immunization with the HisAK70 DNA-vaccine may provide a rapid, suitable, and efficient vaccination strategy to confer cross-protective immunity against VL and CL.

## Background

The leishmanioses cover a range of vector-borne diseases caused by infection with various species of intracellular protozoan parasites of the genus *Leishmania* [1]. Although the burden of the leishmanioses and other neglected tropical diseases (NTD) mainly falls on the poorest areas of the global population [2–4], current studies show that these NTD are adapting to changing environments and spreading into new geographical areas worldwide [5, 6]. Specifically, the leishmanioses have been endemic in southern Europe for centuries [7]. Nevertheless, the northward spread of parasites from the Mediterranean region will depend on whether climate and land cover permit the sand flies vectors to establish seasonal biting rates that match those of southern Europe [5, 8, 9]. Recent published data from a focus of human leishmaniosis have demonstrated that several isolates are representative of a new human-infective *Leishmania* sp. in Ghana [10].

Additionally, *Leishmania infantum* isolates from an endemic area in Spain have been able to infect more than 560 of immunocompetent patients and these parasites have exhibited high virulence in terms of infection index [11]. The most common forms are cutaneous leishmaniosis (CL), which causes skin sores and social stigmatization, and visceral leishmaniosis (VL), which affects several internal organs (spleen, liver and bone marrow) and is potentially fatal if untreated. The leishmanioses represent the second-largest parasitic killer in the world after malaria [12]. There is now an urgent need for an effective vaccine for veterinary and medical prophylaxis [13]. *Leishmania major* in the Old World, and *L. infantum* (= *L. chagasi*) in the Mediterranean region of the Old World and in the Americas [14, 15] are among the main species that are responsible for CL and VL, respectively. Also, canine leishmaniosis is endemic in the Mediterranean basin, and is a public health problem which should be tackled [16, 17].

On another note, there is still no vaccine for use in humans [18], and conventional chemotherapies for the treatment of CL and VL are usually long, expensive and inadequate due to toxicity and resistance [19]. Nevertheless, new approaches from both nanomedicine or inventions related to the cream formulations are very important, avoiding the side effects of drugs during the treatment [20, 21]. Advances in our understanding of *Leishmania* pathogenesis and the generation of host protective immunity in animal models facilitate the development of this urgently needed vaccine [22, 23]. DNA vaccines are not only reasonably simple to manage but also immunogenic and offer a protein similar to the native protein. Furthermore, the induction of Th1 and CD8+ T cell immune responses, a common property of DNA vaccines, is essential to *Leishmania* infection control [22]. Taken together, DNA vaccination against *Leishmania* has been considered a hopeful technology and the development of such a preclinical trial-vaccine (LEISHDNAVAX) for use in humans has been recently tested ex-vivo in human cells and in rodent models of VL and CL [24, 25]. In addition, the novel LEISHDNAVAX-DNA vaccine candidate in combination with a standard antileishmanial drug seems to improve the treatment of the experimental murine VL [26].

The main area of our research interests are the development of suitable protocols both as a prophylactic as well as a therapeutic vaccine against multiple *Leishmania* species using efficient multiantigenic formulations. Recently, the plasmid pCMV-HISA70m2A was generated and used to vaccinate mice against *L. major* infection in our laboratory. We demonstrated that genetic immunization with pCMV-HISA70m2A provided protection in mice against both footpad swelling, and visceralization in the model of CL [27]. In addition, in the present study, the efficiency of the DNA-vaccine has been further enhanced by the inclusion of KMP-11 (kinetoplastid membrane protein-11) antigen (Ag) into the antibiotic-free plasmid pVAX1-asd. We focus on seven *Leishmania* Ag (the four core histones, A2, KMP11 and 70 kDa heat shock protein (HSP70)) that have already been successfully tested as DNA vaccines against CL or VL [22, 27]. The molecule KMP-11 is found in association with membrane structures (at the cell surface, flagellar pocket and intracellular vesicles) [28] in the kinetoplastid protozoa [29] and is highly conserved (>95 % homology) in all *Leishmania* species. Thus, as a special feature considering protein localization sites in the parasite, KMP-11 is both an extracellular and intracellular Ag. Additionally, KMP-11 plays an essential role in the infectivity-virulence (its surface expression is higher in amastigotes than in promastigotes and increases during metacyclogenesis) and other biological features of the parasite [30, 31]. Furthermore, several studies based on DNA vaccination [32–34], peptide-pulsed dendritic cells (DC) [35], or synthetic multi-epitope peptide strategies [36] have shown the efficacy of the immunodominant Ag KMP-11 against experimental CL and VL. The rest of the components of the HisAK70 vaccine are exclusively intracellular Ags. The protective potential of the nucleosomal *Leishmania* histones [37, 38] and H1 [39] have been described. A2 protein is an amastigote specific virulence factor that is required for *Leishmania* parasite survival in a mammalian host and plays a role in the visceralization during VL [40]. Immunization, with the A2 as DNA, offered protection against the invasion of macrophages and disease progression in a murine model of VL [40–42]. Finally, the single HSP70 as DNA failed to confer protection in a murine model of CL [43], whereas administration of the HSP70 gene from the parasite fused with other Ags in a DNA vaccine showed high efficacy against VL [44] and CL [27].

In this study by applying lessons learned from the past, we have enhanced a DNA-vaccine to provide cross-protection against CL and VL. For this purpose, we have cloned the full-length coding sequences of seven *Leishmania* genes, as we mentioned before, into the mammalian expression vector pVAX1 that expresses the resulting polyprotein, called HisAK70, in mammalian cells. The plasmid pVAX1-HisAK70 was used to vaccinate BALB/c mice against both *L. major* and *L. infantum* challenges.

## Methods

### Ethics statement

The animal research described in this manuscript complied with Spanish (Ley 32/2007) and European Union legislation (2010/63/UE). The protocols used were approved by the Animal Care Committee of Complutense University of Madrid (reference number 02/11/10). All procedures and euthanasia were performed under C0_2_ anesthesia, and all efforts were made to minimize suffering.

### Vaccine preparation process

In this study, we followed our previous method [27] to retrieve the sequences of different *Leishmania* genes from the annotated collections of all publicly available DNA sequences (GeneDB and GenBank databases): H2A (Lin J21.V3.1160), H2B (Lin.J09.V3.1410), H3 (LinJ10.V3.0920), H4 (Lin J31.V3.3320), A2 (GenBank S69693), KMP11 (GenBank XM**_**001468996.1), and HSP70 (GenBank CAA69282.1). Subsequently, GeneOptimizer® software generated the optimized sequences [45] that were synthesized chemically (GeneArt) as a single coding region of 4416 bp (**HisAK70)**. This sequence encodes the carboxy-terminal Epitope tag (E-Tag) fusion polyprotein (from N- to C-terminus, H2A-H2B-H3-H4-A2-KMP11-HSP70-*E-Tag*) of 1472 amino acid residues. E-Tag is a short peptide sequence (13 amino acids) useful for the labeling and detection of proteins using western blotting technique. This expression cassette was cloned into the eukaryotic expression plasmid **pVAX1** (Invitrogen) to obtain the recombinant plasmid **pVAX1::HisAK70**.

**Fig. 1:**
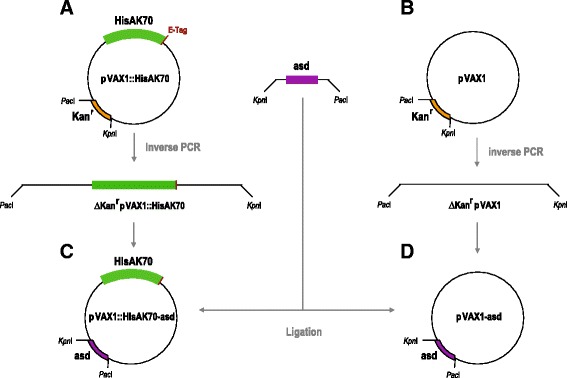
Schematic diagram of the construction of pVAX::HISAK70-asd and pVAX-asd. The kanamycin resistance gene in the eukaryotic expression vectors was replaced by the *asd* gene. **a** To generate pVAX::HISAK70 without kanamycin resistance cassette (Kan^r^), inverse PCR with primers noKan*Pac*I-1R 5’-CTTGTTTAATTAA GCGAAACGATCCTCATCCTGTC-3’ (*Pac*I site underlined) and noKan*Kpn*I-1D 5’-GACGAGGGTACCATTATTAACGCTTACAATTTC-3’ (*Kpn*I site underlined) were employed for outward amplification. The *asd* gene starting at bp 170 and ending at bp 1345 was PCR amplified from bacterial chromosomal with primers asd*Kpn*I-1D 5’ CTGCAAGGTACCCTACGCCAACTGGCGCAGCAT-3’ (*Kpn*I site underlined) and the asd*Pac*I-1R 5’-TTGGCTGTTAATTAAATGGTGAAGGATGCGCCACAG-3’ (*Pac*I site underlined) creating an amplicon with *Pac*I and *Kpn*I sites on its ends. **b** To generate pVAX1 without Kan^r^, inverse PCR with primers noKan*Pac*I-1R and noKan*Kpn*I-1D were employed for outward amplification. **c**–**d** The PCR products of *asd*, ∆kan^r^pVAX1::HISAK70 and ∆kan^r^pVAX1 were each double digested with *Pac*I and *Kpn*I, gel isolated, ligated, and transformed. Thus, pVAX1::HisAK70-asd and pVAX1-asd without resistance gene were obtained

In order to avoid the potential biosafety and clinical hazards derived by using antibiotic resistance genes in DNA vaccines, in this work we followed a previously described system based on the use of the *asd* (aspartate-semialdehyde dehydrogenase) gene as a selection marker to replace the antibiotic resistance markers [46, 47]. Thus, the resistance gene for kanamycin in pVAX1::HisAK70 was replaced by the *asd* gene. We obtained antibiotic-free plasmids **pVAX1::HisAK70-asd** and **pVAX1-asd** as follows (see details in Fig. 1). The *asd* gene (GenBank AE017220) from the genome sequence of *Salmonella enterica* serovar Choleraesuis strain SC-B67 was amplified by PCR and excised (*Kpn*I/*Pac*I). Subsequently, the fragment was purified and then ligated into ∆kan^r^pVAX1::HisAK70 (previously digested with these enzymes). pVAX1-asd was generated following a similar method but using the corresponding template for inverse PCR (pVAX1). Ligation mixtures were transformed into electrocompetent Δ*asd E. coli* cells (mutant strain χ6212 which lacks the *asd* gene) that was kindly provided by Kenneth Roland and Roy Curtiss III at the Biodesign Institute (Arizona State University, USA) [48]. The recombinants were identified on LB agar plates, and the *E. coli* colonies were screened by PCR and sequence analysis.

The expression plasmid pVAX1::HisAK70-asd and empty vector (pVAX1-asd) were purified using the EndoFree plasmid Giga kit (Qiagen, Hilden, Germany) according to the manufacturer’s recommendations. The endotoxin-free DNA plasmids were resuspended in sterile saline solution and were stored at −20 °C until the day of vaccination.

### Transfection of plasmid constructs and Western blotting

The expression of HisAK70 was detected in mammalian cells by transiently transfecting pVAX1::HisAK70-asd into Chinese Hamster Ovary (CHO-K1) cells using Lipofectamine LTX and PLUS Reagent (Invitrogen-lifeTechnologies) according to the protocol (Protocol Pub. No. MAN0007822 Rev.1.0) provided by the manufacturer. CHO-K1 cells were seeded (into 24-well plates at a density of 1.5 × 10^5^ cells per well) in F­12 K Medium (Kaighn’s Modification of Ham’s F­12 Medium, Gibco) with 10 % fetal calf serum (FCS) and 2 mM L-glutamine at 37 °C in 5 % CO2. The CHO-K1 cell cultures were maintained at 90 % confluence and subsequently, they were transfected and incubated for 48 h at 37 °C with 5 % CO_2_. Then, the cells were harvested and lysed by the addition of 40 μL per well of Laemmli’s buffer (Bio-Rad) containing beta-mercaptoethanol. Lysates (15 μL) were resolved by SDS-PAGE (sodium dodecyl sulfate polyacrylamide gel electrophoresis). Protein bands were electrophoretically transferred to a polyvinylidene difluoride (PVDF) membrane (GE Healthcare), which was blocked for 1 h in TBST blocking buffer (Tris-Buffered Saline and Tween 20). To detect the Ag blotted on the membrane, a mouse monoclonal anti-*Leishmania* A2 antibody (Abcam ab150344) was added at an appropriate dilution (1:500) and incubated with the membrane. E-tag was also detected by monoclonal HRP/ Anti-E Tag Conjugate (GE Healthcare Life Sciences) diluted to 1:5000. Because β-tubulin is ubiquitously expressed in all eukaryotic cells, it was used as a loading control for Western blotting assays involving protein detection. Thus, the membrane was probed with a β-tubulin loading control monoclonal antibody (Sigma) at a dilution of 1:5000 for 1 h at room temperature, washed in TBST, and probed with an HRP-conjugated anti-mouse IgG secondary antibody (Sigma) at a dilution of 1:15,000. Detection was performed using a chemiluminescent substrate (Pierce® ECL western Blotting). Immunoreactive bands were detected using the ChemiDoc™ XRS+ System with Image Lab 5.2 Software (Bio-Rad LifeScience).

### Mice, parasites and preparation of soluble Ag

Eight-week-old female BALB/c mice (Harlan Interfauna Ibérica) were maintained at Complutense University of Madrid under standard conditions. *L. infantum* parasites (M/CAN/ES/96/BCN150 zymodeme MON-1) and *L. major* parasites (clone V1: MHOM/IL/80/Friedlin) were maintained as previously described [49]. Soluble *Leishmania* Ag (SLA) was prepared from stationary cultures of promastigotes as previously described [50].

### Vaccine schedule

Two groups of mice (*n* = 30) subcutaneously (s.c.) received 175 μg of pVAX1::HisAK70-asd (**HisAK70**) or pVAX1-asd (**empty vector**) in 40 μL saline in the right footpad on days−60,−45 and−30. In parallel, a group of control mice (*n* = 15) was inoculated with **PBS** alone using the same procedure.

### Infection protocols

To evaluate the vaccine efficacy against VL, fifteen mice of each group were then infected by intravenous injection of 5 × 10^5^ stationary-phase promastigotes of *L. infantum* in 100 μl PBS at day 0. Five mice of each group were euthanized after 28, 42 and 91 days of infection, respectively. After sacrifice, the spleens and livers were removed and subjected to a limiting dilution assay for a parasite load assessment. To evaluate vaccine effectiveness against CL, five mice of each group were infected s.c. in the left footpad with high dose challenge (5 × 10^5^ metacyclic *L. major* promastigotes) in a volume of 30 μL at day 0. Infective-stage promastigotes (metacyclics) were isolated from stationary cultures (5 days old) by negative selection using peanut agglutinin (Vector Laboratories), as previously described before [51]. The course of infection was monitored weekly by measuring footpad swelling with a caliper. Mice were euthanized by cervical dislocation at 5 weeks post-infection (p.i.) because at this time, the lesions from control groups were larger than 4 mm in diameter or showed signs of ulceration, and both parameters were clinical endpoint criteria that we have previously established. Draining lymph nodes (DLN) and spleens were removed from the euthanized mice and subjected to a limiting dilution assay. Additionally, nitric oxide (NO) release, arginase activity and cytokine profiles were determined in both VL and CL experimental models, as described below. Either in VL and CL, the experiments were repeated once to ensure reproducible results.

### Quantification of parasite burden

Parasite burdens in spleens, livers and in the local DLN were determined by a limiting dilution assay [52].

### Isolation of bone marrow-derived DC (BMDC) and coculture with splenocytes or DLN cells

Ten days before euthanasia, bone marrow was harvested from the femurs and tibias of naïve BALB/c mice (*n* = 4) and cultured in the presence of 20 ng/mL murine granulocyte macrophage colony-stimulating factor (GM-CSF; PeproTech, London, UK), as previously described [53]. On day 10, nonadherent cells could be used as DC based on their expression of CD11c. The BMDC exhibited a myeloid DC phenotype and were plated at 1 × 10^6^ cells/mL in 6-well plates and pulsed or not with SLA (50 μg/mL). After 24 h, DC were collected and used for in vitro stimulation of splenocytes or DLN cells as described below. At 28 and 42 days after *L. infantum* infection, or 5 weeks after *L. major* infection, mice were euthanized, and single-cell suspensions of the spleens or DLN were prepared, respectively, and resuspended at a final concentration of 2 × 10^6^ per mL in complete DMEM medium supplemented with 10 % heat-inactivated FCS, 2 mM l-glutamine, 100 U/mL penicillin, and 100 μg/mL streptomycin in 24-well plates. Cell suspensions and BMDC, which had been left unstimulated or pulsed with SLA as described above, were mixed at a ratio of 5:1 and cocultured at 37 °C and 5 % CO_2_.

### NO assay

The concentration of nitrite, which is a byproduct of NO production, was measured in the culture supernatant after 96 h using the Griess assay as described [54].

### Arginase activity assay

In the VL assays, after removing supernatants to measure NO release at 96 h, cells were incubated for 30 min in lysis buffer (0.1 M Tris–HCl, pH 7.5, 300 μM NaCl, 1 μM PMSF, 1 % Triton X-100). Lysates were then assayed for intracellular arginase activity as previously described [55]. One unit of enzyme activity was defined as the amount of enzyme that catalyzes the formation of 1 mmol of urea/min. In the CL assays, arginase activity at the *L. major*-infected footpads was determined using 5–10 mL of footpad homogenate as described elsewhere [27].

### Cytokine analysis

Cells were co-cultured for 96 h, and the culture supernatant was collected and stored at −20 °C. The production of Ag-specific IL-4 (eBioscience), IL-13 (R&D Systems), IFN-γ and IL-10 (Diaclone) was determined by ELISA according to the manufacturers’ suggested protocols.

### Histopathology

At 42 days after infection with *L. infantum*, mouse liver tissues were fixed in fixative solutions and sent to Anapath (Anatomic Pathology Laboratory, Granada, Spain) for sectioning, and H&E (hemotoxylin and eosin) staining. Finally, stained sections were carefully analyzed under the microscope by Dr. Ana Nieto. We scored granulomas as previously described [56–59]: immature (developing granuloma containing infected Kupffer cells), mature (more developed than immature granulomas), or sterile (parasite-free granuloma).

### Statistical analysis

Statistical analyses were performed using SigmaPlot version 11.0 (Systat Software, Inc). Data were assessed for normality and subsequently statistical analyses were determined by a paired Student *t* test. Significant differences were determined and are designated with asterisks as follows: *P <0.05, **P <0.01.

## Results

### HisAK70 protein can be expressed in CHO-K1 cells

**Fig. 2:**
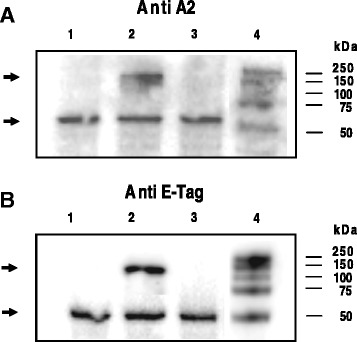
Expression of HisAK70 in CHO-K1 cells after transfection. **a**-**b** HisAK70 protein expression was detected from CHO-K1 transfected cells using specific anti-A2 and anti-E Tag antibodies. Lane 1, total protein extract from empty vector-transfected cells (pVAX1-asd); lane 2, cells transfected with pVAX1::HISAK70-asd; and lane 3, extract from non-transfected CHO-K1 cells. Proteins were separated on a 10 % SDS-PAGE gel. The positions of molecular mass markers are indicated on the right. HisAK70 (152 kDa) and the control protein loading, β-Tubulin (52 kDa), are highlighted by arrows, on the left. Data of one representative out of three independents are given. Relevant portions of each blot are shown

The expression of HisAK70 in CHO-K1 cells transfected with pVAX1::HisAK70-asd was detected using a mouse monoclonal anti-*Leishmania* A2 antibody and E-Tag was also detected by monoclonal HRP/ anti-E Tag Conjugate (Fig. 2).

### HisAK70 vaccination induces protection against *L. infantum* or *L. major* infection in mice

**Fig. 3:**
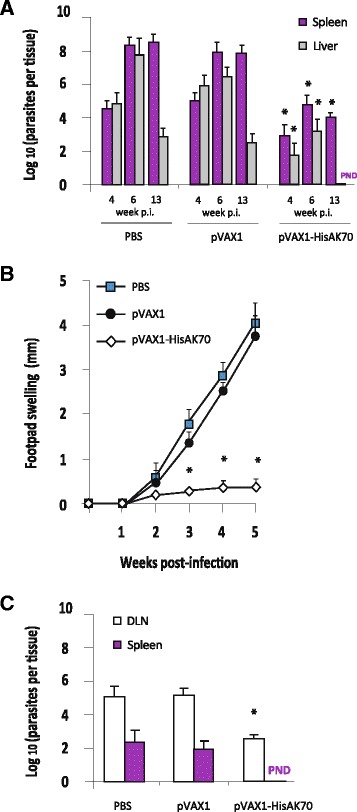
HisAK70 vaccination induces protection against *L. infantum* or *L. major* challenges in mice. **a** Parasite burden was assessed in the spleen and liver at days 28, 42 and 91 after *L. infantum* infection by limiting the dilution assay. **b** Course of *L. major* infection in mice. The mean diameter of induration (± S.D) in the footpad at various times after infection. **c** Mean number of parasites per popliteal DLN and spleen (± S.D) at 5 weeks after *L. major* infection. Data are presented as the mean ± S.D. (*n* = 5). P.N.D., parasites not detected. Asterisks indicate *P* < 0.05 with respect to control mice

We analyzed parasite suppression by comparing each experimental value with the mean control value. As expected, empty plasmid (pVAX-asd) immunization was ineffective in mice, as parasite burdens at various times p.i. were similar to control values, both in the VL and CL experimental models. In the VL experimental model, HisAK70 vaccinated mice had significantly lower parasite burdens (*P < 0.05*). Thus, a 30 % reduction in splenic parasite burden and a 59 % reduction in hepatic parasite burden were achieved in HisAK70 immunized mice after 28 days of infection with *L. infantum*. Later, a 40 % reduction in splenic parasite burden and a 62 % reduction in hepatic parasite burden were achieved in those HisAK70 immunized mice at 42 days p.i.. Finally, after 91 days p.i. a 52 % reduction in parasite burden in spleen and a 100 % reduction in hepatic parasite load were achieved in HisAK70 vaccinated mice (Fig. 3a, *P < 0.05*). In the CL experimental model, other groups of mice were infected with *L. major* parasites in the footpad, and the time course of swelling at the site of infection was evaluated. Figure 3b shows that control groups of mice succumbed to progressive disease. In contrast, vaccinated mice with HisAK70 did not show lesions at the footpad or, in some cases, they had significantly smaller footpad lesions up to 5 weeks after infection with *L. major*, with no signs of ulcer development. Moreover, the numbers of parasites in popliteal-DLN were significantly lower in mice immunized with HisAK70 than those in control mice (Fig. 3c, *P < 0.05*), resulting in a 50 % suppression of parasite numbers. Interestingly, these vaccinated mice did not show the parasite visceralization observed in the control mice (Fig. 3c), resulting in 100 % inhibition of parasite numbers in the spleen.

### HisAK70 vaccination enhances pro-inflammatory/ anti-inflammatory cytokine secretion ratios after experimental infection with *L. infantum* or *L. major*

The antileishmanial cellular immune responses after infections were evaluated by measuring the production of both IFN-γ and the anti-inflammatory cytokines (IL-10 and IL-4). Our data showed that control groups of infected mice did not enhance the IFN-γ/ IL-10 (Table 1) or IFN-γ/ IL-4 (Table 2) ratios in response to SLA-DC pulsed stimulation, as opposed to what was found in the *L. infantum* or *L. major*- infected mice previously vaccinated with HisAK70. Additionally, we found IL-13 levels at day 42 p.i. to be significantly greater (*P* < 0.05) in HisaK70 vaccinated mice compared with levels in *L. infantum* infected control mice (Table 1).

**Table 1:** The arginine metabolism and cytokine production in mice infected with *L. infantum* at 42 days after infection

Groups of mice	μM	mU	Ratio	pg/ml
	Nitrites	Arginase activity	IFN-γ/IL-10	IL-13
PBS	8 ± 3	32 ± 5	3,0	218 ± 46
Empty vector	9 ± 4	23 ± 2	2,6	234 ± 38
HisAK70	21 ± 3 (*)	8 ± 3 (*)	7,5 (*)	396 ± 50 (*)

Data are presented as the mean ± S.D. (*n* = 5). Asterisks indicate *P* < 0.05 with respect to control mice

**Table 2:** The arginine metabolism and cytokine production in mice infected with *L. major* at 5 weeks after infection

Groups of mice	μM	mU	Ratio
	Nitrites	Arginase activity	IFN-γ/IL-4
PBS	10 ± 5	4121 ± 813	2,3
Empty vector	11 ± 4	3130 ± 604	3,1
HisAK70	18 ± 2 (*)	601 ± 250 (*)	5,1 (*)

Data are presented as the mean ± S.D. (*n* = 5). Asterisks indicate *P* < 0.05 with respect to control mice

### HisAK70 DNA vaccination was successful in BALB/c mice and granulomas completed the maturation stage

**Fig. 4:**
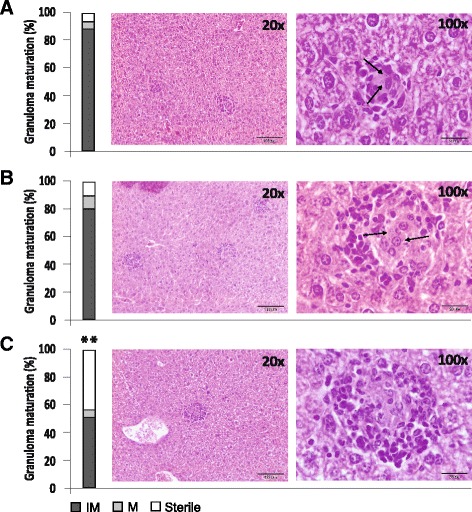
HisAK70 vaccinated mice develop efficient sterile granulomas. Percentage of hepatic granuloma maturation and representative granulomas from H&E stained liver sections at day 42 p.i. in (**a**) PBS, (**b**) empty vector and (**c**) HisAK70 vaccinated mice. Control mice show high amount of immature granulomas, whereas HisaK70 vaccinated mice show well-developed mature and sterile granulomas. Images were acquired at the indicated magnifications and arrows indicate the presence of amastigotes in the granulomas. All data are presented as the representative mean from each experimental group of mice. Asterisks indicate *P* < 0.01 with respect to control mice. IM, immature granuloma; M, mature granuloma; Sterile, parasite-free granuloma

It is well established that in human, canine and various experimental murine (BALB/c and C57BL/6) models of VL, host resistance is strongly associated with efficient granuloma development in the liver [58]. Immature granulomas fail to control infection, but these types of granulomas decrease in number during the course of *L. infantum* infection in BALB/c mice, developing mature and sterile granulomas to eradicate intracellular parasites. Thus, hepatic granulomatous inflammation resolves by 8 weeks p.i., with the majority of parasites cleared [58–60]. As we mentioned before, HisAK70 was successful in vaccinated mice, resulting in 62 % inhibition of parasite numbers in the liver at day 42 p.i. To better characterize the ability of HisAK70 vaccine to anticipate antileishmanial protective response, we assessed granuloma maturation in the livers from *L. infantum* infected mice. Our results showed that in the control mice, infected immature granulomas were predominant (Fig. 4a–b) although some sterile granulomas were also evident. However, the percentage of sterile granulomas observed in HisK70 vaccinated mice at day 42 p.i. increased by approximately 400 % compared with control mice (Fig. 4c). A closer histological examination was performed at 91 days p.i (13 weeks) in our laboratory in a separate experiment and revealed that although the histological profile was similar in *L. infantum* infected mice, control mice were represented by medium and large-size sterile granulomas, whereas HisaK70 vaccinated mice showed sterile granulomas that were significantly smaller in size (Table 3) and had a different cellular composition, characterized by a low amount of Kupffer cells compared to control mice. This evidence would suggest that cells moved out of the granuloma, gradually returning the liver to its pre-infection state in vaccinated mice.

**Table 3:** Percentage of various types of hepatic sterile granulomas in mice infected with *L. infantum* at 42 days after infection

Groups of mice	Small	Medium	Large
PBS	19 ± 11	51 ± 14	38 ± 16
Empty vector	21 ± 14	45 ± 10	34 ± 10
HisAK70	92 ± 12 (*)	8 ± 6 (*)	ND

Data are presented as the mean ± S.D. (*n* = 5). Asterisks indicate *P* < 0.05 with respect to control mice. *N.D* not detected

### Effects of HisAK70 vaccination in the arginine metabolism of phagocytic cells during infection in the VL and CL murine models

Because it has been reported that *Leishmania* interacts with the host cell metabolism via arginase or NO synthase [61], we investigated changes in both enzymatic activities during infection with *L. infantum* (Table 1) or *L. major* (Table 2). As expected, the upregulation of the arginase pathway restricted arginine accessibility of NO synthesis and resulted in low nitrite levels in the control groups of infected mice. Interestingly, HisAK70 vaccination enhanced the ability of *L. infantum* or *L. major*- infected mice to produce NO in response to SLA-DC pulsed stimulation. Additionally, compared to the control groups of mice, HisAK70 vaccinated mice demonstrated dramatically decreased arginase activity at various times after respective infections.

## Discussion

The main goal of the study was to evaluate whether HisAK70, administered as a DNA vaccine, could induce protective immune response against VL and CL. Thus, we employ the well-characterized BALB/c models of *L. infantum* and *L. major* infection. Quantification of parasite burden in different tissues represents the main tool for the analysis of immune responses to experimental leishmaniosis [51]. However, we need to have a clear understanding about which other remarkable markers are associated with host immune protection against leishmaniosis. In this context, previous studies described the methods used to evaluate *L. infantum* and *L. major* infections in mice with regard to the formation of mature granulomas, that provide the microenvironment for intracellular parasite killing in the liver [56, 58–60, 62], and progression of cutaneous lesions at the site of parasite inoculation [63], respectively. In our study, these methods have also been used to evaluate HisAK70 DNA vaccination efficiency.

One of the main safety concerns in the field of DNA vaccine technology, is the use of antibiotic resistance genes present in conventional plasmids and which may have undesirable side-effects. Thus, we followed an alternative strategy based on the use of antibiotic-free host-plasmid balanced lethal systems [46] to select and maintain the recombinant plasmid pVAX1::HisAK70-asd. A major finding of the present study is that this HisAK70 vaccine strategy confers protection against these two *Leishmania* species. Such a finding is particularly relevant in the field of control NTD because *L. infantum* and *L. major* species live in diverse regions on the planet, causing very different forms of disease (visceral and cutaneous forms). Although these two species have distinct geographical distributions and are transmitted by different vector species to different mammalian reservoir hosts, there are some evidences of positive gene flow between *L. infantum* and *L. major*. These hybrid strains were isolated from both the sand fly vector [64] and immunocompromised patients [65].

Our results suggested that HisAK70 enhances antileishmanial immunity at various stages. Immunization with HisAK70 noticeably reduced footpad lesions and parasite burdens relative to infected control mice. It is well known that anti-*Leishmania* immunity is most effectively achieved and maintained by the existence of persistence parasites. For that reason, an efficient vaccine may simply require strengthened immunity against the development of disease rather than provide sterile protection [13, 66]. The induction of cross-protection may be due to diverse components present in HisAK70 vaccine containing epitopes with broad species specificity. Overall, the effectiveness of the HisAK70 vaccine is based on the ability of immunized mice to achieve the control of key factors, such as the ratios iNOS/Arginase activity, IFN-γ /IL-10 or IL-4; a common property previously described in DNA vaccines [22, 27, 67]. Our findings that the anti-*L. infantum* response in HisAK70 vaccinated mice developed a high percentage of mature and sterile granulomas, and was accompanied by significant levels of IL-13 at day 42 p.i. support the notion that this cytokine plays a crucial role in ensuring efficient hepatic granuloma maturation to control parasite load during VL [49, 56]. It should be noted that the control of *L. infantum* infection in HisAK70 vaccinated mice has been confirmed in our laboratory after 91 days p.i. when hepatic parasite burden was fully resolved in these mice, whereas levels of parasites were maintained in control mice. At this point, control mice maintained high chronic parasite burdens in spleen. In contrast, HisAK70 vaccine contributed to a significant reduction in the number of parasites in this tissue. These data indicated that although the lack of parasite clearance in the spleen, HisAK70 immunization is essential to protect the host against parasite growth and responsible for parasite control in this organ during late stages of infection with *L. infantum*.

## Conclusions

We conclude that, despite the main differences in the mechanisms of pathogenesis between these various *Leishmania* species, our data confirm previous evidence that a vaccine against several *Leishmania* species is feasible [67–69] thus HisAK70 may be considered a potential antileishmanial agent. However, improved strategies for the development of HisAK70-based vaccination and immunotherapy have yet to be further explored. In the light of recent studies indicating that a DC-based vaccine in combination with DNA improved the immunogenicity of the DNA vaccine in mice [70], we decided to address the role of HisAK70 in this context. Thus, heterologous prime-boost strategies (HisAK70 pulsed-DC prime-HisAK70-DNA boost and HisAK70-DNA prime- HisAK70 pulsed-DC boost) are being extensively tested in our laboratory in order to determine whether similar strategies can be used to increase vaccine effectiveness. We hypothesize that these approaches, which combine HisAK70 immunization regimens, may represent promising alternatives to induce specific CD4+ and CD8+ T cell responses, and ensuring long-term immunity against leishmaniosis. Future development of these studies to other models (canine) could further demonstrate the potential of HisAK70 vaccine strategy and thereby take another step toward achieving the global control of the leishmanioses.
